# Pharmacological protection from a sugar-rich diet through inhibition of Ras/MAPK signaling

**DOI:** 10.1016/j.isci.2026.117656

**Published:** 2026-09-22

**Authors:** Mihails Laskovs, Jacob S. Cook, Andrew R. Pitt, Corinne M. Spickett, Cathy Slack

**Affiliations:** 1 School of Medicine, Pharmacy and Biosciences & Aston Institute for Membrane Excellence, Aston University, Birmingham B4 7ET, UK; 2 Manchester Institute of Biotechnology, University of Manchester, Manchester M1 7DN, UK; 3 School of Life Sciences, Warwick University, Coventry CV4 7AL, UK

**Keywords:** *Drosophila*, Ras/MAPK signaling, metabolism, lipidomics, lifespan

## Abstract

Consumption of sugar-rich diets adversely affects health and reduces life expectancy through the excessive production and storage of triacylglycerides (TAGs), leading to metabolic dysfunction. Here, we show that in *Drosophila*, pharmacological inhibition of Ras/mitogen-activated protein kinase (MAPK) signaling using the highly specific MEK inhibitor, trametinib, mitigates several of the detrimental effects of a high-sugar diet by reducing TAG accumulation, restoring TAG composition, and increasing survival. Notably, Ras/MAPK activity is required for the transcriptional response to elevated sugar intake, including expression of the glucagon-like hormone, *Akh*. We further demonstrate that adipokinetic hormone (AKH) is essential for the protective effects of trametinib under high-sugar conditions. Together, our findings support Ras/MAPK signaling as an important link between dietary sugar and metabolic dysfunction and show that its pharmacological inhibition can uncouple excess sugar intake from adverse metabolic outcomes.

## Highlights

• Trametinib extends lifespan on both normal and high-sugar diets
• Trametinib prevents TAG accumulation on high-sugar diet
• Transcriptional changes to high-sugar diet are mostly reversed by trametinib
• The protective effects of trametinib on high sugar require the neuropeptide, AKH

## Graphical abstract

**Figure:**
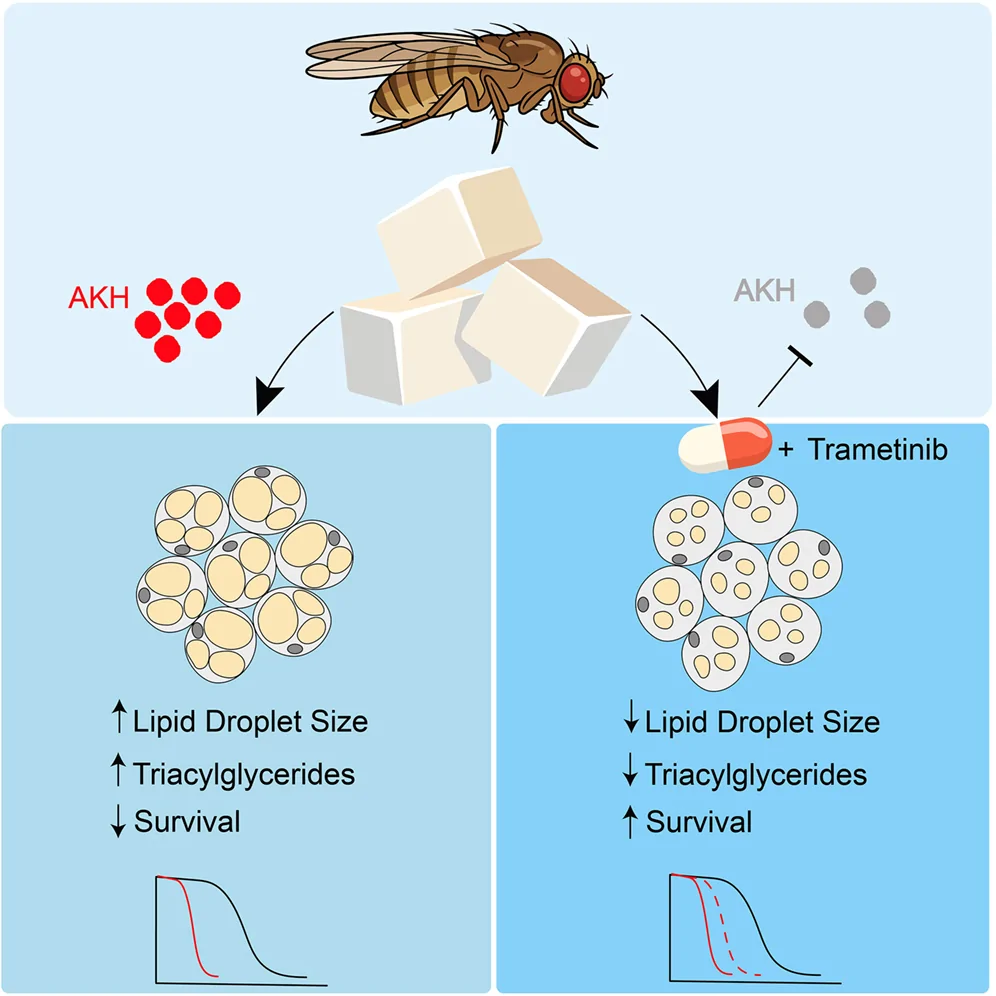

## Teaser

Pharmacology; Molecular biology; Diet

## Introduction

Dietary sugar consumption has increased dramatically over recent decades with added sugar now attributed to around 8% of the total global calorie intake.^1^ Sugar-rich diets are associated with poor health outcomes and are a contributing factor in the development of obesity, diabetes, certain cancers, and cardiovascular disease^2^ as well as accelerated aging.^3^ However, excessive sugar consumption remains prevalent worldwide despite these known health risks, representing a significant societal health burden.

Excess dietary sugar promotes *de novo* lipogenesis, in which surplus carbohydrates are converted into fatty acids. High-sugar intake increases glycolytic flux, producing large amounts of pyruvate and subsequently acetyl-CoA,^4^ which is then used as the two-carbon precursor for fatty acid synthesis. In addition, activation of sugar-responsive transcriptional programs that drive lipogenic gene expression to reprogram metabolism toward lipid synthesis^5^ increases the production and storage of triacylglycerides (TAGs). This accumulation of TAGs, particularly in non-adipose tissues, results in lipotoxicity and impaired metabolic homeostasis.^6^ Given the general reluctance of people to change their dietary habits, interventions that uncouple dietary sugar intake from TAG storage could reduce the metabolic burden of high-sugar diets, but this requires a better understanding of the molecular pathways involved.

Ras/mitogen-activated protein kinase (MAPK) signaling has recently been implicated in the metabolic response to a high-sugar diet through its interactions with key nutrient-sensing pathways, including insulin/IGF-1 signaling (IIS). Elevated dietary sugar can enhance activation of Ras/MAPK signaling to influence cellular growth and metabolism,^7^^,^^8^ and dysregulation of this pathway or its downstream effector, ERK, has been shown to modulate lipid metabolism primarily through control of adipocyte differentiation and metabolic transcriptional programs.^9^^,^^10^^,^^11^^,^^12^^,^^13^ Moreover, high-sugar diets can enhance the growth and malignancy of cells with oncogenic Ras activation,^14^ indicating that sugar availability can modulate Ras-dependent phenotypes. However, a direct role for Ras/MAPK signaling in the metabolic response to high-sugar diet is not clear.

*Drosophila melanogaster* has proved an important model in understanding the interaction between dietary sugar intake and organismal health. Exposure of adult flies to a high-sugar diet induces phenotypes that closely resemble those observed in mammals, including hyperglycemia, insulin resistance, increased lipid accumulation, cardiac dysfunction, and reduced survival.^15^^,^^16^^,^^17^ Coupled with a high degree of evolutionary conservation across key metabolic regulators, *Drosophila* provides a genetically tractable and experimentally malleable system for dissecting the molecular mechanisms that underlie diet-induced metabolic dysfunction.

Here, we show that pharmacological inhibition of Ras/MAPK signaling in *Drosophila* using trametinib, a highly specific small-molecule inhibitor of MEK, suppresses TAG accumulation and improves survival in flies exposed to a high-sugar diet. Interestingly, these beneficial effects of trametinib were not dependent on canonical lipolysis or *de novo* lipogenesis pathways. Lipidomics analysis revealed an overall shift in the fatty acyl (FA) chain length of the TAG species present following trametinib treatment, while transcriptomic analysis identified enrichment of genes involved in metabolic remodeling, including several neuropeptide hormones, suggesting that Ras/MAPK inhibition in the presence of high dietary sugar alters tissue physiology to coordinate the neuroendocrine control of energy utilization and storage. Furthermore, we show that loss of adipokinetic hormone (AKH), a functional analog of mammalian glucagon, is required for the trametinib-dependent effects on TAG levels and survival under excess dietary sugar conditions. Together, our findings implicate Ras/MAPK signaling in the downstream metabolic response to high dietary sugar and demonstrate that pharmacological inhibition of this pathway can uncouple excess dietary sugar from its detrimental downstream effects on lipid metabolism and survival.

## Results

### Trametinib decreases ERK phosphorylation and extends lifespan on both normal and high-sugar diets

Previous studies have demonstrated that ingestion of trametinib, a selective MEK inhibitor, extends *Drosophila* lifespan under normal dietary conditions.^18^^,^^19^^,^^20^ To investigate whether trametinib also provides survival benefits under a high-sugar diet, we analyzed lifespan in response to trametinib treatment when we increased the sugar content of the fly food 8-fold from 5% (w/v) sucrose (1× sugar) to 40% (w/v) (8× sugar). We focused our experiments on females as previous work has demonstrated that the pro-longevity effects of trametinib under normal nutritive conditions are sexually dimorphic, with robust lifespan effects observed in females, but limited or no benefits to male survival.^20^ We first confirmed that trametinib was still able to attenuate Ras/MAPK activity on high-sugar diet by assessing phosphorylation of the downstream signaling effector, ERK. Comparable decreases in the ratios of phospho-ERK to total-ERK were observed following trametinib treatment under both normal and high-sugar conditions (Figure 1A). As has been previously reported, exposure to high dietary sugar shortened adult lifespan (∼55% decrease in median lifespan; Figure 1B; Table S1).^16^^,^^17^^,^^21^^,^^22^ Interestingly, we observed increases in median lifespan on both 1× and 8× sugar diets in trametinib-treated flies compared to untreated controls (Figure 1B; Table S1). We used Cox proportional hazards (CPH) analysis with relevant *a priori* contrasts to analyze the effects of sugar concentration and trametinib treatment as covariates on survival. While both sugar concentration (hazard ratios [HR] = 45.77, 95% confidence interval [CI] 28.78–72.80, *p* < 0.001) and trametinib treatment (HR = 0.55, 95% CI 0.43–0.70, *p* < 0.001) were each significantly associated with survival, no significant interaction was detected (*p* = 0.976), indicating that the effects of trametinib on survival were consistent across both diets (Table S2). Thus, trametinib can effectively inhibit ERK activation and promote positive effects on survival under both normal and high-sugar conditions.

**Figure 1:**
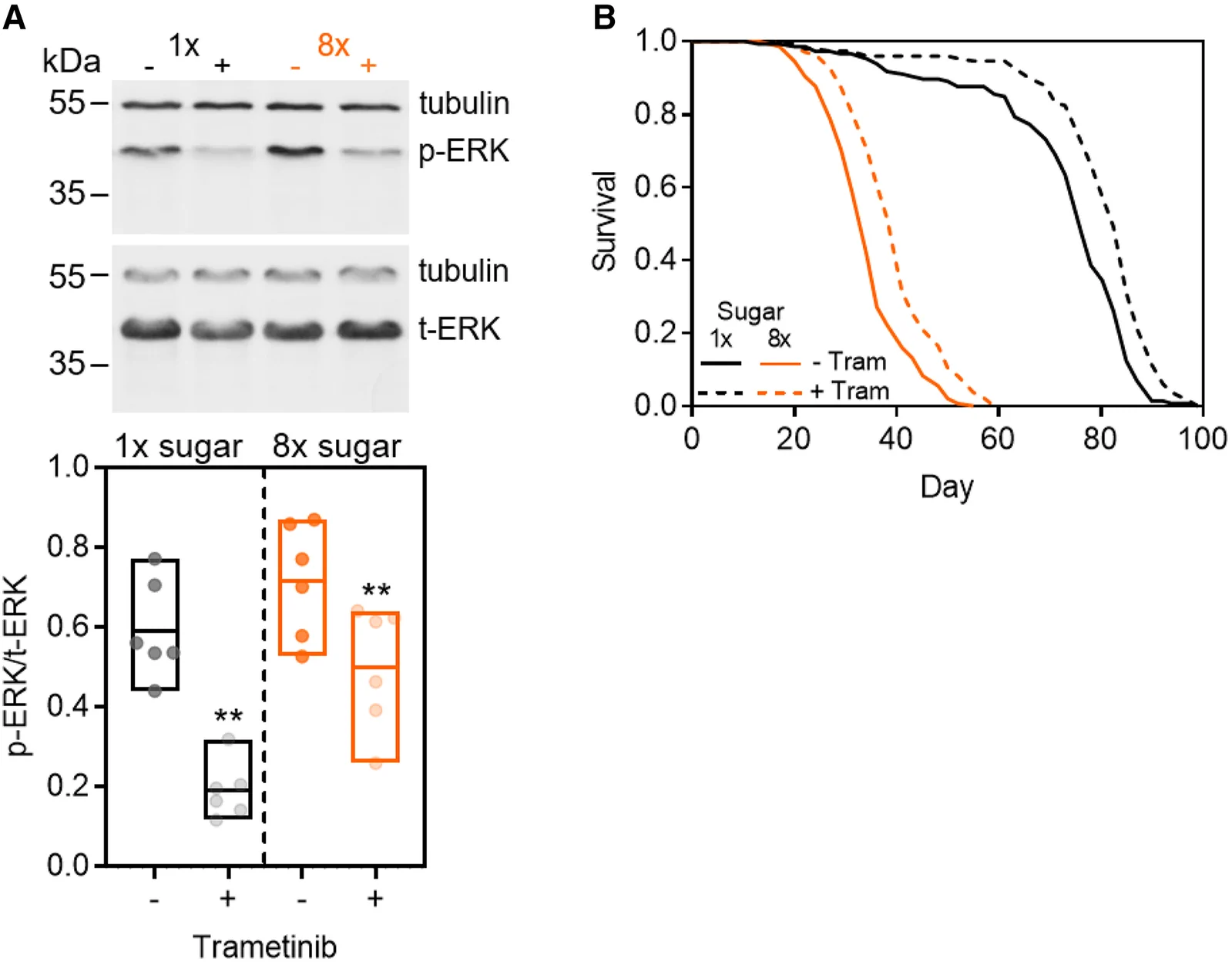
Trametinib inhibits ERK activation and extends lifespan on high-sugar diet (A) Representative western blots and quantification of the ratios of phosphorylated to total ERK in protein extracts from 10-day-old female flies maintained on normal (1×) or high (8×) sugar diets either on vehicle-only (−) or trametinib-containing (+) food, normalized to tubulin. Box plots represent minimum, mean, and maximum with values for individual biological replicates overlaid as points (*n* = 6 biologically independent samples, ANOVA, ∗∗*p* < 0.05, Tukey’s HSD). (B) Survival of female *wDah* flies on normal sugar (1×, black lines) or high sugar (8×, orange lines) containing either vehicle alone (solid lines) or trametinib (dotted lines). (1× sugar − trametinib: *n* = 145 dead/1 censored; 1× sugar + trametinib: *n* = 145 dead/5 censored; *p* = 5.2 × 10^−7^, log rank test; 8× sugar − trametinib: *n* = 144 dead/4 censored; 8× sugar + trametinib: *n* = 133 dead/3 censored; *p* = 1.0 × 10^−7^, log rank test).

### Trametinib attenuates increased TAG stores under high-sugar diet

Exposure of adult flies to a high-sugar diet is associated with several metabolic defects including increased glycogen storage and adiposity. We confirmed that control flies fed a high-sugar diet showed elevated whole-body stores of both glycogen and TAGs (Figures 2A and 2B). While trametinib exposure had no impact on glycogen stores in flies fed either normal or high-sugar diets, it specifically reduced TAG stores in flies fed a high-sugar diet (Figures 2A and 2B). Furthermore, lipid droplet sizes in the adult fat bodies of trametinib-treated flies were significantly reduced on both normal and high-sugar diets compared to control untreated flies (Figures 2C and 2D). We also confirmed that while feeding rates were reduced on high-sugar food, they were comparable for both control and trametinib-treated flies (Figure S1) so the changes in TAG stores we observed could not accounted for by differences in food intake. Together, this suggests that trametinib prevents lipid hypertrophy associated with high-sugar feeding.

**Figure 2:**
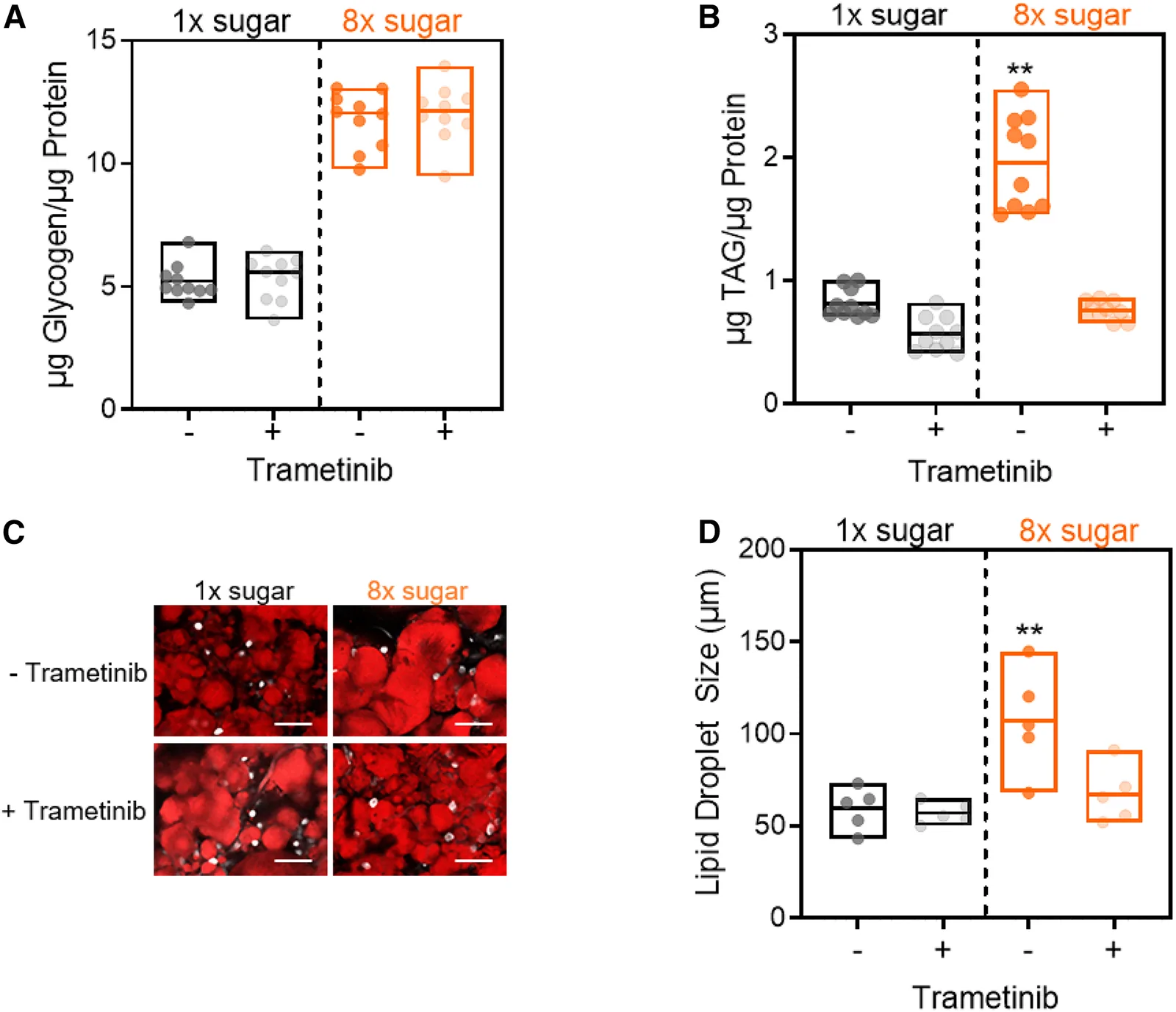
Trametinib inhibits TAG accumulation and reduces lipid droplet size on high-sugar diet (A) Glycogen concentrations in homogenates from 10-day-old female flies maintained on normal (1×) or high (8×) sugar diets, either on vehicle-only (−) or trametinib-containing (+) food, normalized to total protein content. Box plots represent minimum, mean, and maximum values with individual biological replicates overlaid as points (*n* = 10 biologically independent samples). (B) TAG concentrations in homogenates from 10-day-old female flies maintained on normal (1×) or high (8×) sugar diets, either on vehicle-only (−) or trametinib-containing (+) food, normalized to total protein content. Box plots represent minimum, mean, and maximum values with individual biological replicates overlaid as points (*n* = 10 biologically independent samples). ∗∗*p* < 0.001, ANOVA with post hoc Tukey’s HSD. (C) Representative confocal images of lipid droplets (stained with Nile red and nuclei labeled with DAPI in white) in the adult fat bodies of 10-day old female flies maintained on food containing 1× or 8× sugar and either vehicle alone (−Tram) or trametinib (+Tram) (scale bars, 20 μm). (D) Quantification of lipid droplet sizes in adult fat bodies. Box plots represent minimum, mean, and maximum with values for biological replicates overlaid as points (average sizes of 25 lipid droplets per fly, *n* = 5 flies, ∗∗*p* < 0.0001, ANOVA with post hoc Tukey’s HSD).

The reduction in lipid storage in flies fed a high-sugar diet by trametinib could reflect either enhanced lipolysis or impaired lipid synthesis. We therefore genetically manipulated each of these metabolic processes and examined the effects on both survival and TAG storage in response to trametinib treatment. First, we determined the effects of impaired lipolysis using a null allele of *brummer* (*bmm*), which encodes one of the main lipases responsible for the initiation of lipid droplet lipolysis in *Drosophila*.^23^ As has been previously reported, we observed both reduced survival and elevated TAG stores in *bmm* mutant flies fed both normal and high-sugar diets (Figures 3A and 3B). Trametinib treatment not only increased survival but also significantly reduced TAG levels in *bmm* mutant flies regardless of the sugar content of the diet (Figures 3A and 3B).

**Figure 3:**
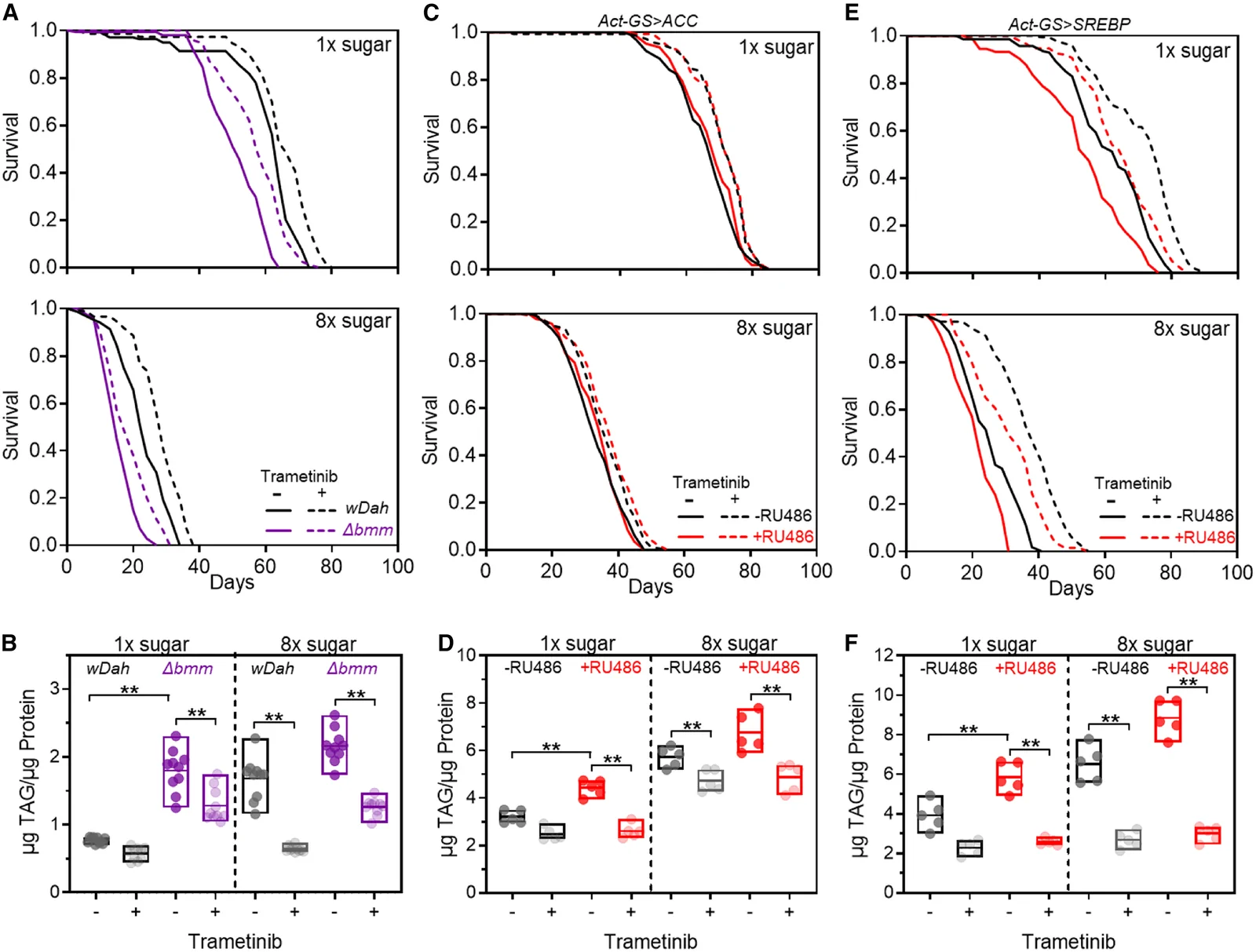
Manipulating the activity of canonical lipolysis or lipogenic pathways does not prevent trametinib-associated lifespan extension or rescue of TAG storage phenotypes on high-sugar diet (A) Survival of female *wDah* (black lines) or *Δbmm* flies (purple lines) on normal sugar (1×) or high sugar (8×), containing either vehicle alone (solid lines) or trametinib (dotted lines). (1× sugar, *wDah* − trametinib *n* = 118 dead/22 censored, *wDah* + trametinib *n* = 129 dead/21 censored, *p* = 4.6 × 10^−7^, log rank test; *Δbmm* − trametinib *n* = 146 dead/4 censored, *Δbmm* + trametinib *n* = 139 dead/10 censored, *p* = 2.1 × 10^−11^, log rank test; 8× sugar, *wDah* − trametinib *n* = 118 dead/22 censored, *wDah* + trametinib *n* = 129 dead/21 censored, *p* = 4.2 × 10^−11^, log rank test; and *Δbmm* − trametinib *n* = 146 dead/4 censored, *Δbmm* + trametinib *n* = 139 dead/10 censored, *p* = 1.5 × 10^−6^, log rank test). (B) TAG concentrations in homogenates from 10-day-old female *wDah* or *Δbmm* flies maintained on normal (1×) or high (8×) sugar diets, either on vehicle-only (−) or trametinib-containing (+) food, normalized to total protein content. Box plots represent minimum, mean, and maximum values with individual biological replicates overlaid as points (*n* = 10 biologically independent samples). ∗∗*p* < 0.05, ANOVA with post hoc Tukey’s HSD. (C) Survival of female *Act*-*GS>UAS*-*ACC* flies on normal sugar (1×) or high sugar (8×) either in the presence (red lines) or absence (black lines) of RU486 (to induce transgene expression) and either vehicle alone (sold lines) or trametinib (dotted lines). (1× sugar, − RU486 − trametinib *n* = 114 dead/18 censored, − RU486 + trametinib *n* = 133 dead/12 censored, *p* = 8.0 × 10^−6^, log rank test; + RU486 − trametinib *n* = 118 dead/10 censored, + RU486 + trametinib *n* = 131 dead/13 censored, *p* = 3.9 × 10^−4^, log rank test; 8× sugar, − RU486 − trametinib *n* = 136 dead/8 censored, − RU486 + trametinib *n* = 143 dead/0 censored, *p* = 1.9 × 10^−3^, log rank test; + RU486 − trametinib *n* = 144 dead/5 censored, + RU486 + trametinib *n* = 140 dead/3 censored, *p* = 1.1 × 10^−5^, log rank test). (D) TAG concentrations in homogenates from 10-day-old female *Act*-*GS>UAS*-*ACC* flies maintained on normal (1×) or high (8×) sugar diets, either in the presence (red) or absence (black) of RU486 containing either vehicle alone (−) or trametinib (+). Box plots represent minimum, mean, and maximum values with individual biological replicates overlaid as points (*n* = 5 biologically independent samples). ∗∗*p* < 0.05, ANOVA with post hoc Tukey’s HSD. (E) Survival of female *Act*-*GS>UAS*-*SREBP* flies on normal sugar (1×) or high sugar (8×), either in the presence (red lines) or absence (black lines) of RU486 (to induce transgene expression) and either vehicle alone (solid lines) or trametinib (dotted lines). (1× sugar, − RU486 − trametinib *n* = 132 dead/18 censored, − RU486 + trametinib *n* = 138 dead/12 censored, *p* = 4.4 × 10^−16^, log rank test; + RU486 − trametinib *n* = 140 dead/10 censored, + RU486 + trametinib *n* = 143 dead/7 censored, *p* = 5.2 × 10^−30^, log rank test; 8× sugar, − RU486 − trametinib *n* = 130 dead/12 censored, − RU486 + trametinib *n* = 127 dead/13 censored, *p* = 2.6 × 10^−68^, log rank test; + RU486 − trametinib *n* = 132 dead/15 censored, + RU486 + trametinib *n* = 134 dead/8 censored, *p* = 2.4 × 10^−19^, log rank test). (F) TAG concentrations in homogenates from 10-day-old female *Act*-*GS>UAS*-*SREBP* flies maintained on normal (1×) or high (8×) sugar diets, either in the presence (red) or absence (black) of RU486 containing either vehicle alone (−) or trametinib (+). Box plots represent minimum, mean, and maximum values with individual biological replicates overlaid as points (*n* = 5 biologically independent samples). ∗∗*p* < 0.05, ANOVA with post hoc Tukey’s HSD.

We then used transgenic overexpression of either *acetyl*-*coA carboxylase* (*acc*), encoding a rate-limiting enzyme in the lipid biosynthetic pathway, or *sterol regulatory element*-*binding protein* (*SREBP*), a key sugar-responsive transcriptional regulator of lipogenesis, to enhance lipid synthesis. We used the RU486-inducible GeneSwitch system for adult-onset ubiquitous overexpression of both transgenes using the *Actin5C* GeneSwitch driver (*Act*-*GS*) and again examined their effects on survival and TAG storage on both normal and high-sugar food in combination with trametinib treatment. Overexpression of both ACC and SREBP led to increased TAG stores under normal dietary sugar conditions, indicative of enhanced lipid biosynthesis (Figures 3D and 3F). These changes in TAG stores were associated with negligible effects on survival for ACC overexpression, while SREBP overexpression caused a small but significant reduction in lifespan (Figures 3C and 3E). Trametinib exposure significantly increased the lifespan of flies overexpressing ACC or SREBP on both normal and high-sugar diets (Figures 3C and 3E) and also reduced TAG stores in these flies under both dietary regimes (Figures 3D and 3F). Moreover, these effects of trametinib on lifespan extension and TAG storage were reproducible with independent transgenes and in flies carrying the *Act*-*GS* driver alone (Figures S2 and S3). Together, these data suggest that the beneficial metabolic effects of trametinib on high-sugar diet are not dependent on changes to the activity of canonical lipolytic or lipogenic pathways.

### Lipidomic analysis reveals changes in both phospholipids and TAGs following trametinib exposure

To further investigate changes in lipid composition in response to trametinib treatment on both normal and high-sugar diets, we extracted total lipids from whole flies for analysis by liquid chromatography-tandem mass spectrometry (LC-MS/MS). We identified a total of 347 lipids across 5 lipid classes, including 88 glycerolipids (GLs), 224 glycerophospholipids (GPs), 12 sphingolipids (SPs), 14 fatty acyls (FAs), and 9 glycolipids (Figure 4A). The most predominant lipids present were TAGs, contributing to over 97% of the lipidome, followed by phosphatidylcholines (PCs) and phosphatidylethanolamines (PEs), which together comprised around 2% of the total lipids present (Figure 4B) based on normalized abundance. Principal component analysis (PCA) revealed that while samples from each treatment group clustered together, there was some overlap between flies fed normal and high-sugar diets in the absence of trametinib. In contrast, we observed substantial separation in the lipidomes of flies on the two respective diets when treated with trametinib (Figure 4C). Thus, the major changes in lipid composition with excess dietary sugar appeared to result from treatment with trametinib.

**Figure 4:**
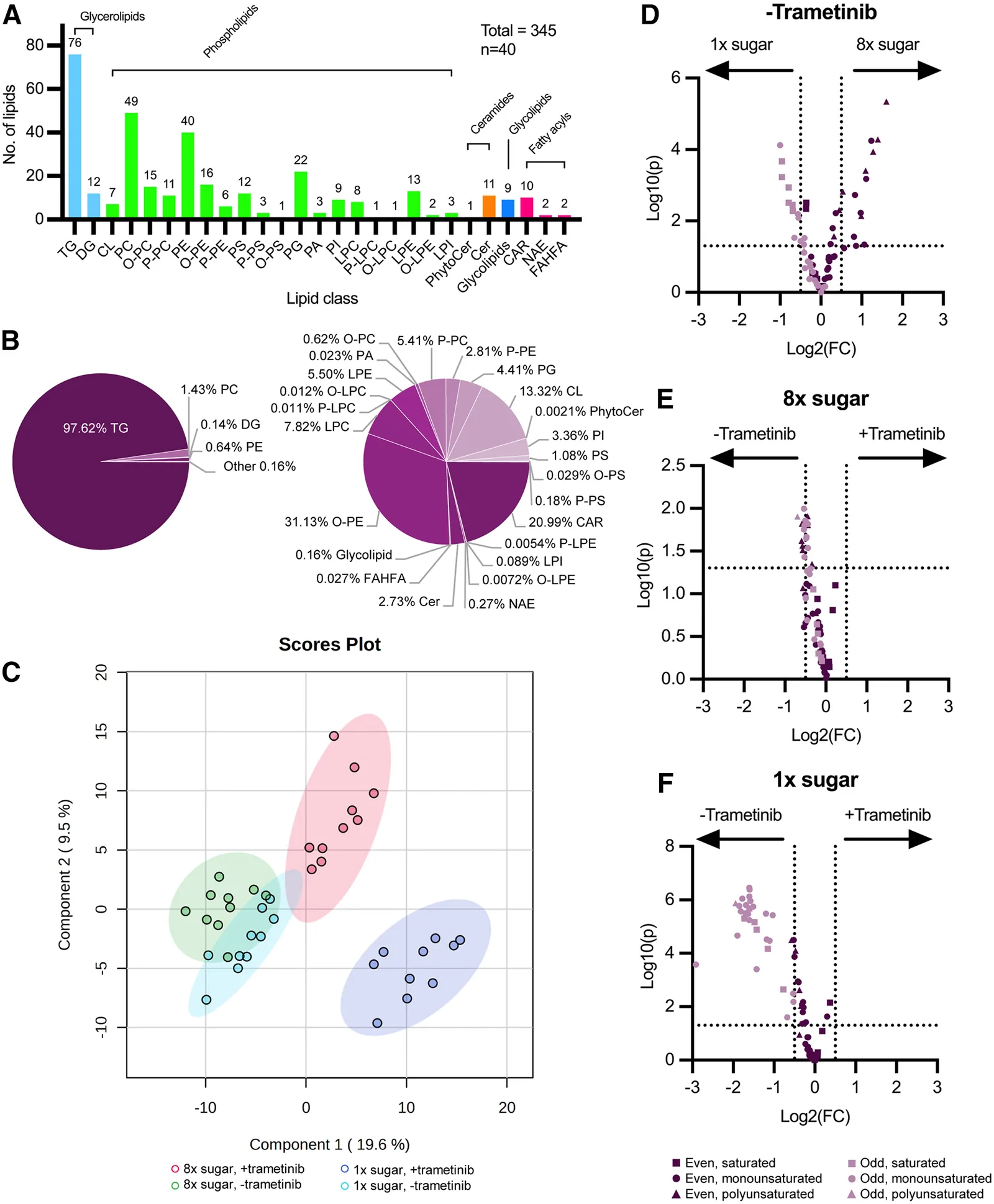
Lipidomics analysis reveals trametinib-dependent shifts in TAG composition under high-sugar diet (A) The number of individual lipid species (*y* axis) detected by LC-MS/MS across multiple lipid subclasses (*x* axis) in all analyzed samples (*n* = 10 biological replicates). (B) Distribution of lipids by normalized abundance showing TAGs as the most abundant species and, separately, the lipid profile of the non-TAG lipid component. (C) PCA plot derived from Lipostar showing discrimination between lipidomic analysis of 1× and 8× sugar diets in the presence or absence of trametinib. (D–F) Volcano plots of the changes in individual TAG species in *Drosophila* extracts under different diet and treatment conditions (significance set at *p* < 0.05 and fold change > 1.5): effect of 1× or 8× sugar diet on flies without trametinib treatment (D); effect of trametinib in flies on 8× sugar diet (E); and effect of trametinib in flies on 1× sugar diet (F).

We saw no overall differences in the normalized abundance of total TAGs in response to high-sugar diet nor in trametinib-treated versus untreated flies even on high-sugar diet (Figure S4), even though we had seen a reduction in TAG stores by biochemical assay. As TAGs were also the major constituent of the lipidome in our LC-MS/MS analysis, we investigated changes to individual TAG species in more detail. Similar to what has been previously reported, we observed that with high-sugar diet there was an increase in even-numbered FA chains and depletion of odd-numbered FA chains (Figure 4D). Trametinib exposure under high-sugar conditions mostly reversed these effects on FA chain length with limited evidence of increased even-chain FAs and little impact on odd-chain FAs despite the flies feeding on a high-sugar diet (Figure 4E). Under normal sugar conditions, trametinib treatment predominantly decreased odd-chain FAs (Figure 4F). Thus, while we observed no overall change in the abundance of total TAGs with trametinib treatment, there was a shift in the chain length of the FAs present depending on the dietary sugar content.

### Transcriptomic analysis of gene expression changes in response to trametinib exposure

To investigate the molecular basis for the metabolic effects of trametinib, we compared genome-wide transcriptional changes in response to trametinib treatment on both normal and high-sugar diets using RNA sequencing (RNA-seq). PCA demonstrated close alignment between replicate samples, which clustered based on nutritional condition and trametinib treatment (Figure 5A). The expression differences between groups could be attributed to two principal components (PC1 and PC2), which together explained ∼66% of the total variation across datasets. A relatively large number of genes (1,181) were significantly differentially regulated in response to high-sugar diet (5% false discovery rate [FDR], Figure 5B), and comparisons to published datasets identified a significant overlap in these sugar-responsive genes to those reported by others^21^ (Figure S5). Surprisingly, very few genes were differentially regulated in response to trametinib treatment under normal dietary sugar conditions (Figure 5B). In contrast, trametinib exposure under high-sugar conditions elicited a much larger transcriptional response with 2,781 genes differentially expressed (Figure 5B).

**Figure 5:**
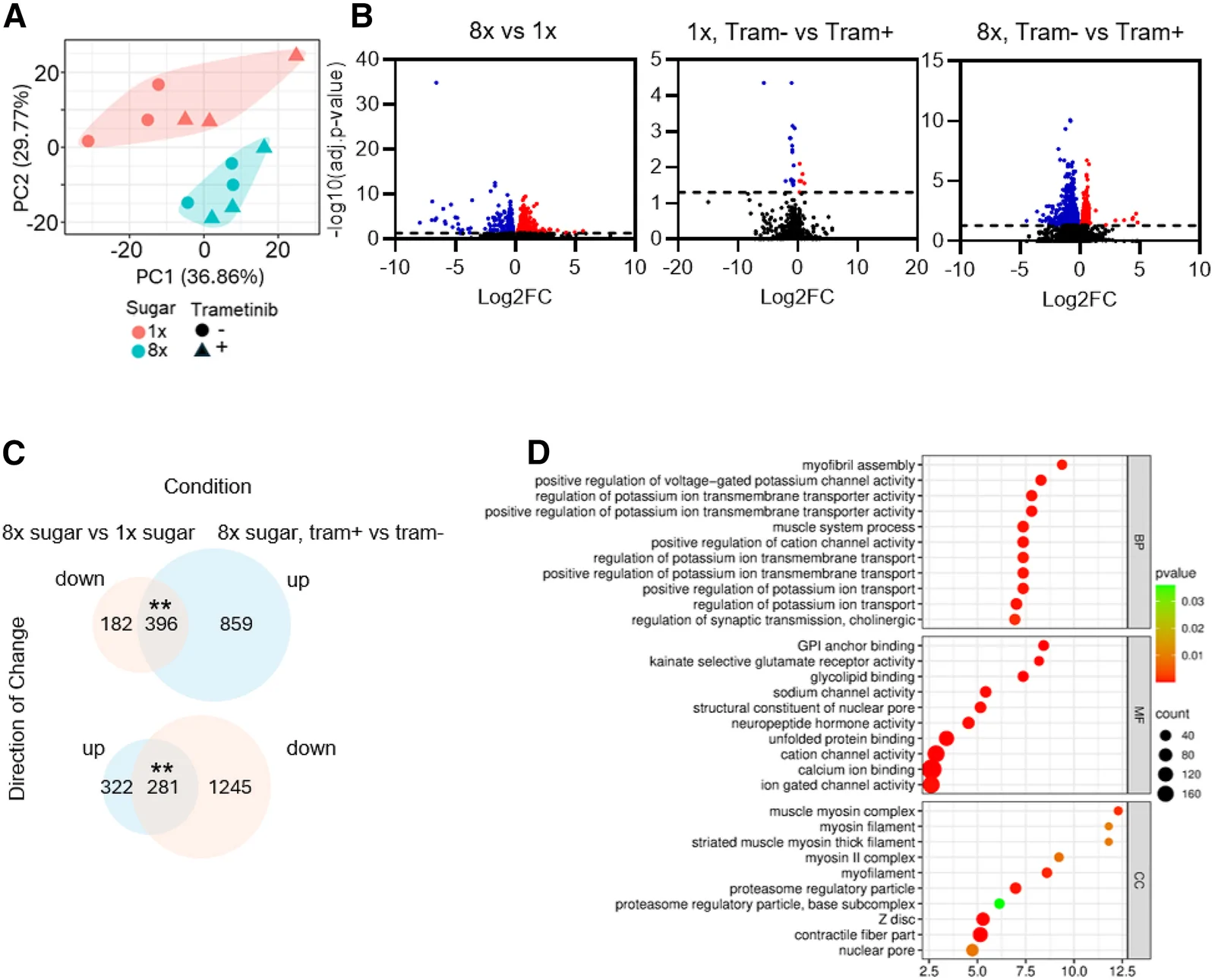
Trametinib-induced gene expression changes on normal and high-sugar diets (A) PCA of RNA sequencing data from 10-day-old adult females maintained on the indicated sugar concentrations in either the absence (−) or presence (+) of trametinib (*n* = 3 biological replicates per condition). (B) Volcano plots showing the number of differentially expressed genes (FDR 0.05) between the indicated conditions (8× sugar vs. 1× sugar, no trametinib; 1× sugar, + trametinib vs. − trametinib; and 8× sugar, + trametinib vs. − trametinib). Each dot represents an individual gene. Downregulated genes are shown in blue and upregulated genes are in red. Horizontal black dotted line denotes adjusted *p* value threshold of −log10(0.05). (C) Proportional Venn diagrams showing the genes that are transcriptionally upregulated in response to high sugar in the absence of trametinib (8× sugar vs. 1× sugar) or downregulated in response to trametinib in flies fed high-sugar diet (8× sugar, − trametinib vs. + trametinib). The overlap is composed of the genes that require Ras/MAPK activation for upregulation in response to high-sugar feeding. The reciprocal situation is shown in the proportional Venn diagram below of genes that are transcriptionally downregulated in response to high sugar in the absence of trametinib (8× sugar vs. 1× sugar) or upregulated in response to trametinib in flies fed high-sugar diet (8× sugar, tram + vs. tram −), and the overlap represents the genes that require Ras/MAPK activation for downregulation in response to high-sugar feeding. Probability of overlap being significantly larger than expected by chance was calculated based on hypergeometric distribution [∗∗*p* < 0.001]). (D) Gene ontology (GO) enrichment analysis of the 677 Ras/MAPK-dependent sugar-responsive genes. Bubble plot shows the top 10 GO terms identified in these genes against a background list of all genes differentially expressed in response to high sugar.

We hypothesized that those genes—or at least a subset of those genes—that showed differential expression in response to high-sugar diet may require Ras/MAPK signaling activity for their activation or repression. We therefore compared all sugar-responsive transcripts to those that were changed in the opposite direction when flies were treated with trametinib while maintained on a high-sugar diet (Figure 5C). Interestingly, we found highly significant overlaps between the genes with both increased and decreased expression in response to high-sugar diet and those that were treated with trametinib in that same dietary sugar context. Thus, Ras/MAPK activation is required for a large proportion of the gene expression changes induced by high sugar.

We further annotated those 677 Ras/MAPK-dependent sugar-responsive genes using Gene Ontology (GO) enrichment analysis (Figure 5D). This revealed a strong overrepresentation of terms associated with muscle structure and contractile function, as well as multiple enriched terms reflecting regulation of membrane excitability. Together, these data indicate that high-sugar diet is associated with gene expression changes indicative of a coordinated remodeling of the cellular contractile architecture of muscle and its electrophysiological properties—effects of which are generally more or less reversed by inhibition of Ras/MAPK activity by trametinib.

We were particularly interested in the enriched category of “neuropeptide hormone activity” as neuroendocrine signaling in *Drosophila* has a well-established role in regulating fat metabolism by integrating nutrient availability with energy expenditure, primarily through the fat body. Among the Ras/MAPK-dependent sugar-responsive genes were those encoding the neuropeptides *Akh*, *Mip*, *Nplp1*, *Dms*, *Dh31*, *itp*, *sNPF*, and *Pdf*, which all showed increased expression in flies fed with a high-sugar diet and which was reversed in the presence of trametinib.

### AKH contributes to the beneficial effects of trametinib on a high-sugar diet

In *Drosophila*, circulating sugar levels are not sensed directly by the insulin-producing cells (IPCs) but rather by the AKH-producing cells of the corpora cardiaca.^24^ AKH functions similarly to mammalian glucagon, binding to a G protein-coupled transmembrane receptor on target cells to initiate an intracellular signaling cascade to increase lipolysis, glycogenolysis, and production of trehalose in the insect fat body.^25^ We first confirmed changes in expression of *Akh* mRNA in response to trametinib by RT-qPCR. Similarly to what we observed in our RNA-seq analysis, *Akh* transcript expression was unchanged by trametinib treatment on normal sugar, increased in response to high sugar, but was restored to expression levels comparable to those under normal sugar conditions by trametinib treatment (Figure 6A). We then assessed the impact of trametinib treatment on lifespan and TAG storage in *Akh* null mutant flies. Interestingly, trametinib was still able to extend the lifespan of *Akh* mutant flies on normal sugar diet but failed to cf. any benefits to their survival on high-sugar diet (Figure 6B). Consistent with these effects on lifespan, trametinib was also unable to reduce TAG levels in *Akh* mutants on high-sugar diet, although elevated TAG levels caused by *Akh* loss-of-function on normal sugar diet were reduced by trametinib (Figure 6C). Thus, AKH activity is required for the beneficial effects of trametinib to reduce TAG accumulation and increase survival under a sugar-rich diet but is dispensable for the effects of trametinib on lifespan under a normal dietary sugar regime.

**Figure 6:**
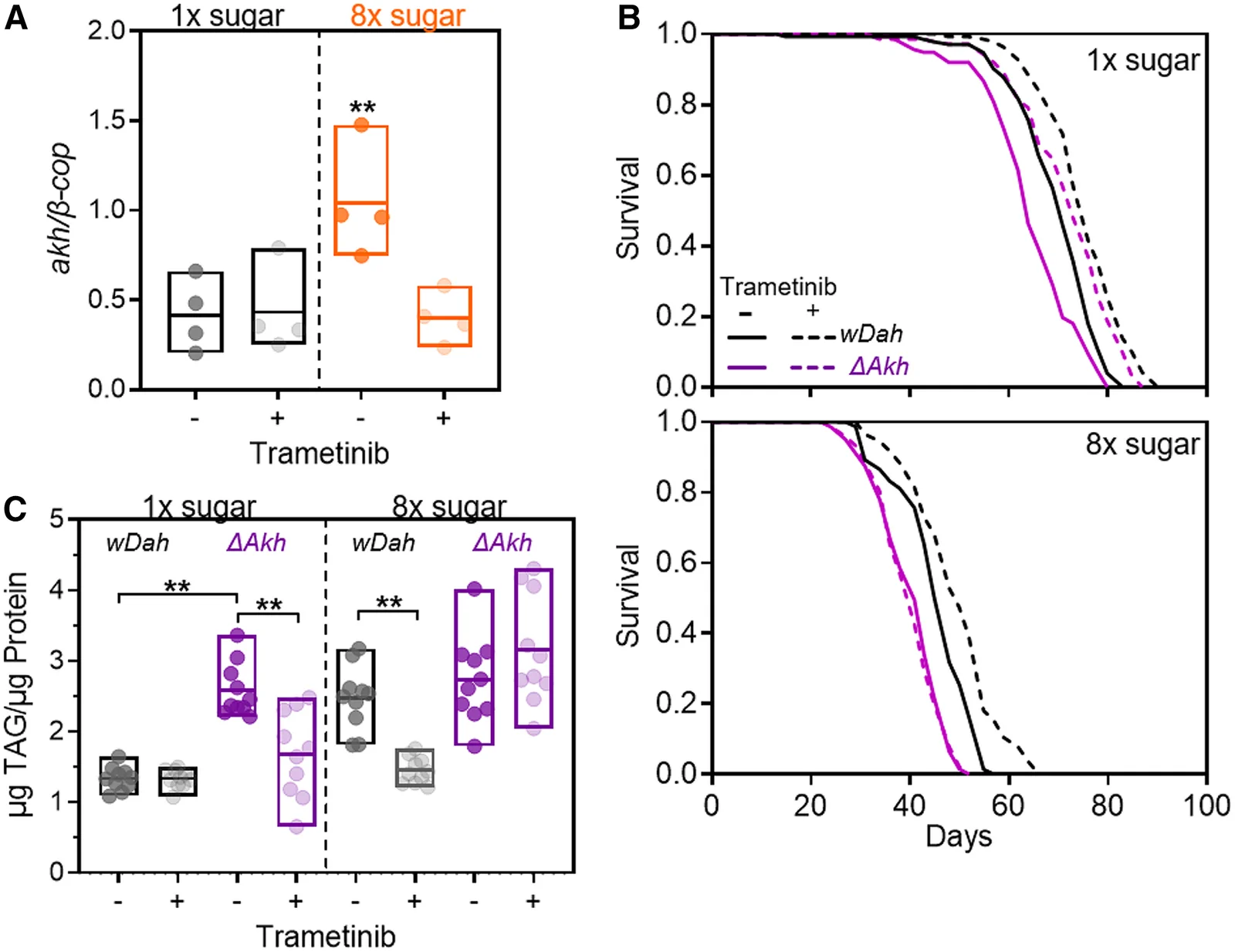
Akh is required for trametinib-mediated lifespan extension and TAG reduction on high-sugar diet (A) Relative *akh* transcript levels measured by RT-qPCR for total RNA extracted from whole 10-day-old female flies maintained on normal (1×) or high (8×) sugar diets, either on vehicle-only (−) or trametinib-containing (+) food, normalized to the expression of *β*-*cop*. Box plots represent minimum, mean, and maximum with individual biological replicates overlaid as points (*n* = 4 biologically independent samples, ∗∗*p* < 0.05, *t* test). (B) Survival of female *wDah* (black lines) or *ΔAkh* flies (purple lines) on normal sugar (1×) or high sugar (8×) containing either vehicle alone (solid lines) or trametinib (dotted lines). (1× sugar, *wDah* − trametinib *n* = 130 dead/20 censored, *wDah* + trametinib *n* = 133 dead/11 censored, *p* = 3.6 × 10^−9^, log rank test; *ΔAkh* − trametinib *n* = 131 dead/7 censored, *ΔAkh* + trametinib *n* = 131 dead/14 censored, *p* = 1.4 × 10^−12^, log rank test; 8× sugar, *wDah* − trametinib *n* = 144 dead/6 censored, *wDah* + trametinib *n* = 144 dead/6 censored, *p* = 1.8 × 10^−7^, log rank test; *ΔAkh* − trametinib *n* = 143 dead/7 censored, *ΔAkh* + trametinib *n* = 145 dead/5 censored, *p* = 0.757, log rank test). (C) TAG concentrations in homogenates from 10-day-old female *wDah* or *ΔAkh* flies maintained on normal (1×) or high (8×) sugar diets, either on vehicle-only (−) or trametinib-containing (+) food, normalized to total protein content. Box plots represent minimum, mean, and maximum values with individual biological replicates overlaid as points (*n* = 10 biologically independent samples). ∗∗*p* < 0.05, ANOVA with post hoc Tukey’s HSD.

## Discussion

Consuming excess dietary sugar negatively impacts on health and is an important contributor to metabolic disease, thereby limiting life expectancy.^26^ Mechanisms to reduce the metabolic burden of high-sugar diets provide an effective means to improve health outcomes given the prevalence of poor dietary habits. Our study demonstrates that in *Drosophila*, pharmacological inhibition of Ras/MAPK signaling using trametinib mitigates several detrimental consequences of a high-sugar diet. The evolutionary conservation of this signaling pathway makes it highly likely that inhibition of Ras/MAPK activity would also offer metabolic benefits in response to high-sugar diet in mammals.

Ras/MAPK signaling has recently emerged as an important longevity regulator under normal dietary conditions. Direct inactivation of Ras orthologs extends lifespan in yeast,^27^ while genetic inhibition of Ras and other downstream pathway components extends lifespan in *Drosophila*.^18^ Furthermore, pharmacological inhibition of MEK activity using trametinib extends lifespan in both *Drosophila* and mice.^18^^,^^19^^,^^28^ We have shown that trametinib also confers lifespan benefits in *Drosophila* under metabolic challenge from excessive dietary sugar. High-sugar diets are known to reduce lifespan across species^21^ and in *Drosophila*, excess dietary sugar recapitulates several aspects of metabolic syndrome observed in mammals, including hyperglycemia, insulin resistance, and increased adiposity.^15^ Our data show that under high dietary sugar conditions, the survival benefits of trametinib are accompanied by reduced TAG storage, suggesting a potential link between these effects.

Trametinib was able to attenuate Ras/MAPK activity on both normal and high-sugar diets, as demonstrated by a visible reduction in the phosphorylation of ERK, the downstream effector of this conserved signaling pathway. Interestingly, while we observed a small increase in ERK phosphorylation by western blot of whole-fly protein extracts in response to high dietary sugar, this was not statistically significant. Previous studies have shown elevated levels of phosphorylated ERK within isolated larval fat bodies in response to high sugar.^7^ We therefore suggest that ERK activation in response to high dietary sugar is likely to be spatially restricted and therefore difficult to detect in whole-animal lysates.

In the present study, we have focused our analyses on the metabolic consequences of trametinib treatment exclusively in female flies based on earlier observations that the lifespan response to trametinib exposure is sexually dimorphic.^20^ Under normal dietary conditions, female flies show robust lifespan extension to trametinib treatment, while for male flies, the lifespan response is minimal or absent altogether. However, whether this sexual dimorphism is maintained under conditions of metabolic stress remains unknown. High-sugar diets may induce metabolic changes that influence the contribution of Ras/MAPK signaling to metabolic homeostasis, and so determining whether Ras/MAPK inhibition similarly protects male flies from the metabolic consequences of a high-sugar diet, or whether the response remains sex-specific, will be an important direction for future studies.

The metabolic effects of trametinib exposure under high-sugar conditions were surprisingly specific to lipids. While high dietary sugar increased glycogen stores, there were no obvious differences in glycogen levels between untreated and trametinib-treated groups. In contrast, both TAG levels and lipid droplet sizes within the fat bodies of trametinib-treated flies were restored to those observed under normal dietary sugar conditions. Interestingly, lipidomic analysis by LC-MS/MS further revealed substantial alterations in TAG composition rather than large changes in overall TAG abundance. In alignment with previous studies, TAGs remained the dominant lipid class after high-sugar feeding (Figure S4) but exhibited shifts in fatty acyl (FA) chain composition with increased prevalence of even-numbered FA chains and a concomitant decrease in odd-numbered FA chains.^29^ These effects of high-sugar diet on FA chain number were effectively reversed by trametinib exposure. Remodeling of FA chain length and composition within TAGs in *Drosophila* can influence lipid droplet dynamics, affecting lipid droplet size, stability, and mobilization,^30^^,^^31^^,^^32^ as well as the availability of lipid-derived signaling molecules that regulate systemic metabolism.^33^ Thus, trametinib may counteract the metabolic effects of high-sugar diet through remodeling of FA composition.

Similar changes to constituent FA content in *Drosophila* in response to excess dietary sugar have been previously attributed to diet-dependent changes in the composition of gut microbiota.^29^ High-sugar diets deplete overall gut commensals,^22^ and as enteric bacteria produce odd-chain FAs, this is thought to drive the sugar-dependent shift in odd- to even-FA chain length.^29^ Trametinib may therefore restore the complement of TAGs under high-sugar feeding to that representative of normal dietary sugar through alterations to the gut microbiota. Interestingly, previous reports have linked the pro-longevity effects of trametinib with protective effects on intestinal physiology under normal dietary conditions.^20^ Trametinib decreases epithelial dysplasia and prevents tumor formation to maintain intestinal integrity during aging,^20^ phenotypes that are often linked with dysbiosis of the gut microbiota.^34^

Interestingly, the ability of trametinib to influence both TAG storage and lifespan was not mediated through canonical lipolytic or lipogenic pathways. Genetic loss of *brummer*, encoding a major lipase that mediates the initiation of lipid droplet breakdown, or overexpression of the key lipogenic factors, ACC and SREBP, was unable to prevent the reduction in TAG stores or extended lifespan observed after trametinib treatment on high-sugar diet. SREBP is a central transcriptional regulator of sugar-induced lipogenesis in both mammals and *Drosophila*, and *ACC* is one of its key target genes.^5^ Notably, we did not observe any significant changes in the expression of *ACC* or other direct SREBP target genes, including *FASN*, *ACS*, *Men*, or *CG6295*, within our transcriptomics analysis. Our results therefore indicate that inhibition of Ras/MAPK signaling affects lipid metabolism through alternative regulatory processes.

Further exploration of the transcriptional response to trametinib revealed that there were strikingly minimal transcriptional changes with trametinib treatment under normal dietary conditions compared to flies maintained on a high-sugar diet. Thus, the downstream physiological effects of trametinib treatment in the absence of nutritive stress are likely mediated through post-transcriptional mechanisms. Previous studies in *Drosophila* have shown that trametinib impairs the activity of RNA polymerase III (Pol III), implicating trametinib as translational inhibitor,^20^ while in mice, trametinib has been shown to reduce the activity of lipogenic enzymes through transcription-independent regulation of chaperone-mediated autophagy.^35^ In contrast, many genes whose expression changed in response to high dietary sugar were regulated in the opposite direction following trametinib treatment, indicating that Ras/MAPK activity is required for a substantial portion of the sugar-responsive transcriptional program. Together, this suggests that Ras/MAPK inhibition produces context-dependent cellular responses according to the nutritional state of the animal.

Notable transcriptional changes under high-sugar feeding that were reverted by exposure to trametinib included several genes encoding metabolically active neuropeptide hormones, among which was *Akh. Akh* encodes the *Drosophila* functional analogue of mammalian glucagon.^24^ AKH is released from the corpora cardiaca in response to high-energy demands and acts mainly on the fat body to mobilize stored energy reserves, including TAGs. We found that genetic loss of AKH activity was sufficient to prevent the beneficial metabolic effects of trametinib under high-sugar conditions. Thus, loss of AKH abolished the ability of trametinib to both reduce TAG accumulation and extend lifespan when flies were fed excess sugar. Interestingly, the pro-longevity effects of trametinib were still observed under normal dietary conditions in *Akh* mutants, indicating that this requirement for AKH activity is specific to metabolic context.

Previous studies have shown that AKH activates ERK, a key downstream effector of Ras/MAPK signaling, via a CaMKII-dependent pathway to promote peroxisomal gluconeogenesis.^36^ Based on our findings, we propose that ERK may also act upstream to regulate AKH-dependent systemic responses to dietary sugar, pointing to a novel bidirectional AKH-ERK signaling axis in metabolic control. Our data therefore highlight an important interaction between Ras/MAPK signaling and neuroendocrine regulation of metabolism and suggest that modulation of endocrine signaling may represent an important mechanism through which Ras/MAPK inhibition reshapes metabolic responses to dietary sugar excess. Targeting this signaling axis could therefore be leveraged to provide a therapeutic strategy for mitigating the metabolic burden associated with excessive sugar consumption.

### Limitations of the study

Pharmacological inhibition of Ras/MAPK signaling using trametinib produces systemic effects on signaling and so does not identify the specific tissues in which Ras/MAPK signaling contributes to the metabolic effects of a high-sugar diet. Future studies employing tissue-specific genetic manipulation of Ras/MAPK signaling will therefore be important to define the cellular sites of action underlying these effects. Finally, all experiments were performed in adult female *Drosophila*, based on previous reports that trametinib robustly extends lifespan in females but has limited effects in males under standard dietary conditions. Whether Ras/MAPK inhibition similarly protects male flies from the metabolic consequences of a high-sugar diet remains an important question for future investigation.

## Resource availability

### Lead contact

Requests for further information and resources should be directed to and will be fulfilled by the lead contact, Cathy Slack (cathy.slack@warwick.ac.uk).

### Materials availability

This study did not generate new unique reagents.

### Data and code availability

• Raw data files from RNA-seq experiments are available from ArrayExpress as E-MTAB-17017 and are publicly available as of the date of publication.
• The metabolomics data have been deposited to the MetaboLights^37^ repository with the study identifier MTBLS14468 and are publicly available as of the date of publication.
• Raw data from Figures 1A, 2C, and 2D were deposited on Mendeley Data at https://data.mendeley.com/datasets/5pxznxt262/1https://doi.org/10.17632/5pxznxt262.1.
• This paper does not report original code.
• Any additional information required to reanalyze the data reported in this paper is available from the lead contact upon request.

## Acknowledgments

This work was supported by the 10.13039/501100000268Biotechnology and Biological Sciences Research Council (10.13039/501100000268BBSRC) through the Midlands Integrative Biosciences Training Partnership (MIBTP). C.S. acknowledges funding from the 10.13039/501100000288Royal Society (RG170325). J.S.C. and C.M.S. acknowledge funding from Aston University College of 10.13039/100018696Health and 10.13039/100019139Life Sciences and The Dudley Group NHS Foundation Trust for a 50:50 PhD studentship. Stocks obtained from the Bloomington *Drosophila* Stock Center (NIH P40OD018537) were used in this study. We also thank S. Gronke, L. Partridge, and R. Kühnlein for *Drosophila* stocks.

## Author contributions

Conceptualization, C.S.; methodology, M.L., J.S.C., and C.S.; investigation, M.L., J.S.C., C.M.S., and C.S.; data analysis, M.L., J.S.C., and C.S.; writing – original draft, M.L. and C.S.; writing – review and editing, C.M.S. and C.S.; funding acquisition, C.M.S. and C.S.; supervision, A.P., C.M.S., and C.S.

## Declaration of interests

The authors declare no competing interests.

## STAR★Methods

### Key resources table

**Table:** 

REAGENT or RESOURCE	SOURCE	IDENTIFIER
**Antibodies**		

rabbit monoclonal phospho-ERK (Thr202/Tyr204)	Cell Signaling	Cat# 4370; RRID: AB_2315112
rabbit monoclonal total-ERK	Cell Signaling	Cat# 4695; RRID: AB_390779
mouse anti-beta tubulin	Abcam	Cat# ab108342; RRID: AB_10866289
goat anti-Rabbit IgG H&L (HRP)	Abcam	Cat# ab6721; RRID: AB_955447
goat anti-mouse IgG H&L (HRP)	Abcam	Cat# ab6789; RRID: AB_955439

**Chemicals, peptides, and recombinant proteins**		

Trametinib	Insight Biotechnology	Cat# HY-148507
Nile Red	Sigma Aldrich	Cat# N3013
Fluoroshield with DAPI	Merck	Cat# F6057
RU486	Cambridge Biosciences	Cat# CAY10006317
TRIzol^TM^ Reagent	Thermo Fisher Scientific	Cat# 15596026
Triglyceride Infinity Reagent	Thermo Fisher Scientific	Cat# TR22421
Triglycerides Liquid	Sentinel Diagnostics	Cat# 17624H
Pierce^TM^ 660 nm protein assay reagent	Thermo Fisher Scientific	Cat# 22660
Infinity Glucose Reagent	Thermo Fisher Scientific	Cat# TR15421
SuperScript II Reverse Transcriptase	Thermo Fisher Scientific	Cat# 18064014
PrecisionPLUS qPCR master mix	PrimerDesign	Cat# Z-PPLUS-SY

**Deposited data**		

RNA-sequencing data	ArrayExpress	ArrayExpress: E-MTAB-17017
Lipidomics data	MetaboLights	MetaboLights: MTBLS14468
Original western blot images	This paper	Mendeley Data: https://data.mendeley.com/datasets/5pxznxt262/1; doi: https://doi.org/10.17632/5pxznxt262.1
Original microscopy images	This paper	Mendeley Data: https://data.mendeley.com/datasets/5pxznxt262/1

**Experimental models: Organisms/strains**		

*D. melanogaster: w*^*Dah*^	This lab/Linda Partridge	N/A
*D. melanogaster: w*^*1118*^	Bloomington *Drosophila* Stock Center	RRID: BDSC_3605
*D. melanogaster: Actin5C-GeneSwitch*	Ford et al.^38^	N/A
*D. melanogaster: bmm*^1^	Grönke et al.^23^	N/A
*D. melanogaster: UAS-Acc*	Bloomington *Drosophila* Stock Center	RRID: BDSC_63224 and RRID: BDSC_63225
*D. melanogaster: UAS-SREBP*	Bloomington *Drosophila* Stock Center	RRID: BDSC_38396
*D. melanogaster: Akh*^*[A ]*^	Galikova et al.^24^	N/A

**Oligonucleotides**		

Akh-F 5′-AAAGCGGGCTGAATCGAAGT-3′	MWG Eurofins	N/A
Akh-R 5′- GATTGCACGAAGCGGAAGA-3′	MWG Eurofins	N/A
β-COP-F 5′-TCTCCGAAAATG GACTTGG-3′	MWG Eurofins	N/A
β-COP-R 5′- GACACCGAGTT CCGTCAAAT -3′	MWG Eurofins	N/A

**Software and algorithms**		

Fiji	ImageJ	Schindelin et al.^39^
Microsoft Excel	Microsoft	https://www.microsoft.com/en-gb
Jmp14	SAS	https://www.jmp.com/en/home
Prism 10.0	Graphpad	https://www.graphpad.com/features
R and R studio (version R-4.2.2)	The R Project for Statistical Computing	https://www.r-project.org/
Adobe Photoshop	Adobe	https://www.adobe.com/uk/products/photoshop.html?promoid=PC1PQQ5T&mv=other
LASX	Leica	https://www.leica-microsystems.com/products/microscope-software/p/leica-las-x-ls/

### Experimental model and study participant details

#### Animals

All experiments were conducted on adult female flies using the *Drosophila melanogaster* background strain, *white Dahomey* (*w*^*Dah*^), which was derived by backcrossing the *w*^*1118*^ mutation into the outbred wild-type *Dahomey* background. *Dahomey* was collected in 1970 in Dahomey (now Benin) and has since been maintained in large population cages with overlapping generations on a 12 h:12 h light to dark cycle at 25°C. *w*^*1118*^ was obtained from the Bloomington *Drosophila* Stock Center (RRID: BDSC_3605). *w*^*Dah*^ carries the endosymbiont *Wolbachia pipientis.* Other *Drosophila* stocks used in this study include *Actin5C-GeneSwitch* (*Act-GS)*,^38^ *bmm*^1^^23^ *UAS-Acc*^40^ (RRID:BDSC_63224 and RRID:BDSC_63225), *UAS-SREBP*^41^ (RRID:BDSC_38396) and *Akh*^*[A ]*^.^24^ All stocks were backcrossed for at least six generations into the outbred *w*^*Dah*^ background, except for *UAS-Acc* and *UAS-SREBP*, which were used as hybrids.

Stocks were maintained and experiments conducted at 25°C on a 12 h light/dark cycle, at constant 60% humidity. Flies were reared on standard sugar-yeast-agar food (1*x* SYA) containing 5% (w/v) sucrose (Tate & Lyle), 10% (w/v) brewer’s yeast (MP Biomedicals) and 1.5% (w/v) agar (Merck).^42^ For high sugar food, the sugar content was increased 8-fold to 40% (w/v). For trametinib treatment, the food was supplemented with 15.6 μM trametinib (Insight Biotechnology) dissolved in DMSO. For GeneSwitch experiments, RU486 (Cambridge Biosciences) dissolved in ethanol was added to the food at a final concentration of 200 μM. For drug-free control treatments, equivalent volumes of the appropriate vehicles were added.

### Method details

#### Lifespan measurements

Flies were housed at a density of 15 flies per vial and transferred into fresh vials every 2–3 days, during which the numbers of deaths and censors were scored. Flies were censored if they were damaged during handling, escaped, or became stuck to the food.

#### Feeding assay

Fly feeding rates were assayed using a proboscis extension assay as previously described.^43^ 10-day old adults (*n* = 15 vials per condition, each vial containing 5 flies) were observed for proboscis extension every 2–3 min over 90 min. Observations were performed blind.

#### Triglyceride assay

Whole fly triacylglyceride (TAG) levels were measured from frozen flies (5 flies per sample) homogenized in 0.05% (v/v) Tween 20. Samples were incubated at 70^o^C for 5 min and supernatants assayed using Triglyceride Infinity Reagent (TR22421, ThermoFisher Scientific) or Triglycerides Liquid (17624H, Sentinel Diagnostics) against a glycerol standard curve. Protein content of homogenates was determined using the Pierce^TM^ 660 nm protein assay reagent (22660, Thermo Fisher Scientific).

#### Glycogen assay

Whole fly glycogen levels were measured from frozen flies. 5 flies per replicate sample were homogenized in 50 μL 0.2 M Na_2_CO_3_. Samples were then incubated at 95°C for 2 h before the addition of 30 μL 1 M acetic acid and 120 μL 0.2 M Na acetate (pH 5.2). 0.0096 units of amyloglucosidase from *Aspergillus niger* (Merck) were added before incubation at 57°C overnight. Glycogen standards of known concentration were similarly processed in parallel. Liberated glucose was then measured using the Infinity Glucose reagent (TR15421, ThermoFisher Scientific) according to the manufacturer’s instructions.

#### Quantification of lipid droplet size

Live flies were anaethetised on ice and dissected in ice-cold PBS. The ventral cuticle was cut away from the abdomen, and internal organs removed leaving the intact abdominal fat body attached. Samples were fixed in 4% (v/v) methanol-free formaldehyde in PBS for 30 min at room temperature followed by 3 × 10 min washes in PBS. Fat bodies were then incubated in Nile Red (0.5 mg/mL; Sigma Aldrich, N3013) followed by 3 × 10 min washes in PBS before mounting in Fluoroshield containing DAPI (Merck). Fat bodies were imaged using a Leica SP8 Lightning confocal microscope and LASX (Leica) software. Lipid droplets were traced manually and measured using Fiji within ImageJ. Representative images were processed in LASX and Adobe Photoshop (Adobe) for publication.

#### Western blotting

Five whole adult flies were homogenized in 100 μL Laemmli buffer containing 100 mM DTT (1,4-Dithiothreitol) and proteins were denatured at 85°C for 10 min ∼20 μg of proteins were separated using 10% SDS–PAGE gels and transferred onto a 0.45-μm nitrocellulose membranes. Primary antibodies used were phospho-ERK (Thr202/Tyr204) (1:1000 dilution, Cell Signaling #4370), total-ERK (1:1000, Cell Signaling #4695) and anti-beta tubulin (diluted 1:10,000; Abcam #ab108342). HRP-conjugated secondary antibodies – either goat anti-rabbit IgG (1:10,000 dilution; Abcam #ab6721) or goat anti-mouse IgG (1:10,000 dilution; Abcam #ab6789) were used. Blots were developed using the ECL detection system (Bio-Rad). Signals were captured using a G:BOX gel documentation system (SynGene) and band intensities were quantified using Fiji in ImageJ.^39^

#### Mass spectrometric analysis of lipids

Lipids were isolated from whole adult *Drosophila* by methanol/methyl *tert*-butyl ether (MTBE) extraction using a protocol adapted from.^44^ Briefly, five 10-day old adult females per sample were homogenized in methanol then lipids extracted in MTBE and water using a volumetric ratio of 10 parts MTBE:3 parts methanol:2.5 parts water. Extracted lipids were lyophilised under oxygen-free nitrogen gas and stored in glass vials at −80°C. Lipids were resuspended in 200 μL of isopropanol prior to analysis.

Samples were analyzed using an ACQUITY UPLC I-Class PLUS System (Waters) coupled to a SELECT SERIES Cyclic ion mobility separation mass spectrometry system (Waters) using reverse phase liquid chromatography tandem mass spectrometry. Samples were injected using partial loop onto an ACQUITY Premier CSH C18 Column, 1.7 μm particle size, 2.1 × 100 mm (55°C), and were eluted during an 18-min gradient using a mixture of solvent A (60:39:1 acetonitrile:water:1M ammonium formate with 0.1% formic acid) and B (90:9:1 isopropanol:acetonitrile:1M ammonium formate with 0.1% formic acid); gradient program consisted of Initial conditions 85 A:15 B, then 70 A:30 B at 2 min, 52 A: 48 B at 2.5 min, 18 A: 82 B at 11 min, 1 A: 99 B at 11.5 min until 15 min, where the gradient returned to 85 A: 15 B until 18 min to allow for column re-equilibration before the next sample was injected. Ten μL/min Leucine-Enkephalin (200 ng/mL) was infused once every minute as the lockspray.

In positive ion mode, capillary, cone and source offset voltages were set to 2V, 40V and 20V, respectively. Source temperature was 120°C and desolvation temperature was 280°C. Cone gas was set to 40 L/h and desolvation gas set to 600 L/h. Nebuliser gas was set to 6 bar, and reference capillary was set to 3 kV. In negative ion mode, capillary, cone and source offset voltages were set to 4V, 27V and 5V respectively. Source temperature was 120°C and desolvation temperature was 300°C. Cone gas, desolvation gas, nebuliser gas, and reference capillary settings were the same as for positive ion mode. The TOF was operated in V mode, with acquisition range 50–2000 m/z. Scan times of MS and MS/MS were 1 s and 0.1s, respectively. MS/MS was acquired using data-dependent acquisition, fragmenting the 5 most intense peaks with an intensity count of >1,000. A dynamic exclusion list was applied, preventing re-fragmentation of ions of the same m/z within 10 s. Fragmentation was performed using collision induced dissociation through a ramp of 25V–45V, after quadrupole isolation using a low mass resolution of 4.7 and a high mass resolution of 15.0.

Samples were resuspended in 200 μL IPA, and for TAG analysis, were diluted 1 in 100 in IPA before 5 μL was injected. To allow for less abundant, more polar lipids to be analyzed, a method that diverted the eluate to waste before TAGs eluted (9.5 min) was used. For this experiment, samples were left undiluted, and 5 μL and 10 μL were injected in positive ion and negative ion modes, respectively. Samples were kept at 10°C in the sample manager of the LC system during analysis, to prevent degradation of lipids and evaporation of solvents. A series of blanks (100% isopropanol) and QCs (a mixture of all samples) were used throughout the batch to manually monitor performance of the instrument. 3 blanks were injected at the start of the batch, and were also injected every 3 sample runs, as well as before and after each QC, while QCs were injected every 10 sample runs. Samples were injected in a randomised order. The experiment was repeated with a new randomised order, to ensure repeatability of findings.

Mass spectra files (.raw) were imported into Lipostar (Molecular Discovery).^45^ Peaks were selected using a signal threshold of 200 in MS and 100 in MS/MS. Peaks obtained in initial blank samples were subtracted from the experimental samples. M/z values of Leu-Enk were used for correction of peak m/z values, using reference values of 556.2771 in positive ion mode and 554.2615 in negative ion mode. M/z values of peaks were compared against the Lipidmaps Structural Database, for hits within 10ppm of the database value. Only identifications with at least 1 fragment within MS/MS, whose m/z matched that of the hypothetical fragmentations within 20ppm, were approved. Kendrick Mass Defect plots were then used to map the retention time for each class of lipid. Integrated peak areas for approved lipids were then exported for analysis. Peak intensities for each lipid were uploaded to Metaboanalyst for Partial Least Squares Discriminant Analysis (PLS-DA) of entire fly lipidomes after pareto scaling. Volcano plots of TAGs were generated in Prism using a minimum *p*-value of 0.05 and Log2FC > 0.5.

#### RNA-sequencing

Total RNA was extracted from 10-day old frozen flies using TRIzol. cDNA libraries were prepared and sequencing (150 bp paired-end) performed by Novogene Co. Ltd (UK) using Illumina NovaSeq platforms. Reads were assessed for quality using FastQC and reads below the minimum quality threshold Q < 10 were discarded. Orphaned reads after filtering were also discarded. Reads were mapped to the Berkeley *Drosophila* Genome Project (BDGP) *Drosophila melanogaster* genome assembly release 6 (Ensembl release v51) using RNA STAR (version 2.7.11b). Reads that were uniquely aligned to annotated genes were counted with FeatureCounts (version 2.1.1). Differential expression analysis was performed using DESeq2 within iDEP (version 2.3.5), Significance was determined using the standard DESeq2 FDR cutoff of adjusted *p* < 0.05. Gene overlaps were visualised using proportional Venn diagrams in BioVenn^46^ and analyzed by hypergeometric analysis in Excel based on the modified Fisher Exact *p*-value test. Gene ontology (GO) analysis was performed using GOrilla.^47^

#### Quantitative RT-PCR

RNA was converted to cDNA using random hexamers and SuperScript II Reverse Transcriptase (Invitrogen). Quantitative PCR of cDNA was performed using 2*x* PrecisionPLUS qPCR master mix (PrimerDesign) on a LightCycler 480 system (Roche). Expression levels of target sequences were calculated using the relative standard curve method and normalised to the reference gene, *β-COP* (FBgn0008635) (Wat et al., 2020). The suitability of *β-COP* as a reference gene for this study was confirmed using Normfinder (https://www.moma.dk/software/normfinder). Primers for quantitative PCR were Akh-F 5′-AAAGCGGGCTGAATCGAAGT-3′, Akh-R 5′- GATTGCACGAAGCGGAAGA-3′, β-COP-F 5′-TCTCCGAAAATGGACTTGG-3′, and β-COP-R 5′- GACACCGAGTTCCGTCAAAT -3′.

### Quantification and statistical analysis

Sample sizes (n) were determined based on previous publications using similar experimental strategies. No statistical method was used to predetermine n. The exact value and the definition of n for each experiment are provided in the corresponding figure legends. No specific methods were used to randomly allocate samples to groups. Data distributions were assumed to be normal, but this was not formally tested. No data were excluded from the analysis. Data collection and analysis were carried out in an unblinded fashion unless otherwise stated. Statistical analyses were mainly performed in R studio (R v3.5.5), Prism (v10.0, Graphpad) or Jmp (v14, SAS). Survival data were analyzed using Log rank test in Excel (Microsoft) or Cox proportional hazards tests in R using the ‘survival’ package (Terry Therneau, https://CRAN.R-project.org/package=survival). The statistical details of experiments can be found in the figure legends including the statistical tests used. For RNA-seq analyses, false discovery rate (FDR) were applied to adjust for multiple testing where FDR <0.05 were considered significant. For other experiments, *p* < 0.05 was considered significant and is indicated by ∗∗ within the figures.
