# Supplementary material for: Pharmacological protection from a sugar-rich diet through inhibition of Ras/MAPK signaling
Source: iScience. 2026 Sep 22;29(10):117656. doi: 10.1016/j.isci.2026.117656 (PMC13657105; doi:10.1016/j.isci.2026.117656)
Supplement: Supplementary file 1 [file mmc1.pdf]

iScience, Volume *29*

Supplemental information

Pharmacological protection
from a sugar-rich diet through inhibition
of Ras/MAPK signaling

Mihails Laskovs, Jacob S. Cook, Andrew R. Pitt, Corinne M. Spickett, and Cathy Slack

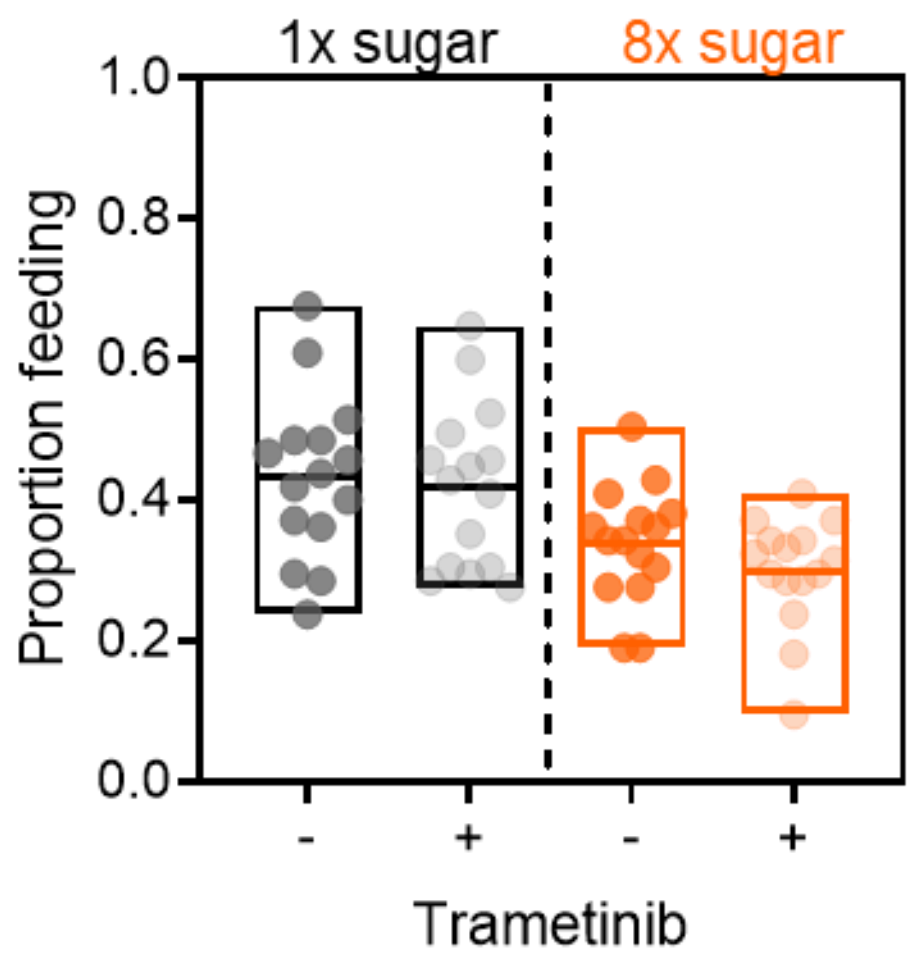

Supplementary Figure S1. Trametinib does not affect feeding behaviour on normal or high sugar diets, related to Figure 1.

Feeding events as a proportion of total events observed per vial for *wDah* females on normal (1x) or high (8x) sugar food containing vehicle alone (-Trametinib) or 15.6 μM trametinib (+Trametinib). Box plots represent minimum, mean and maximum values with individual biological replicates overlaid as points (15 vials per condition, 5 flies per vial). Data were analysed using a Generalized Linear Model with binomial distribution and overdispersion parameter. There was no significant effect of trametinib on feeding (p = 0.76).

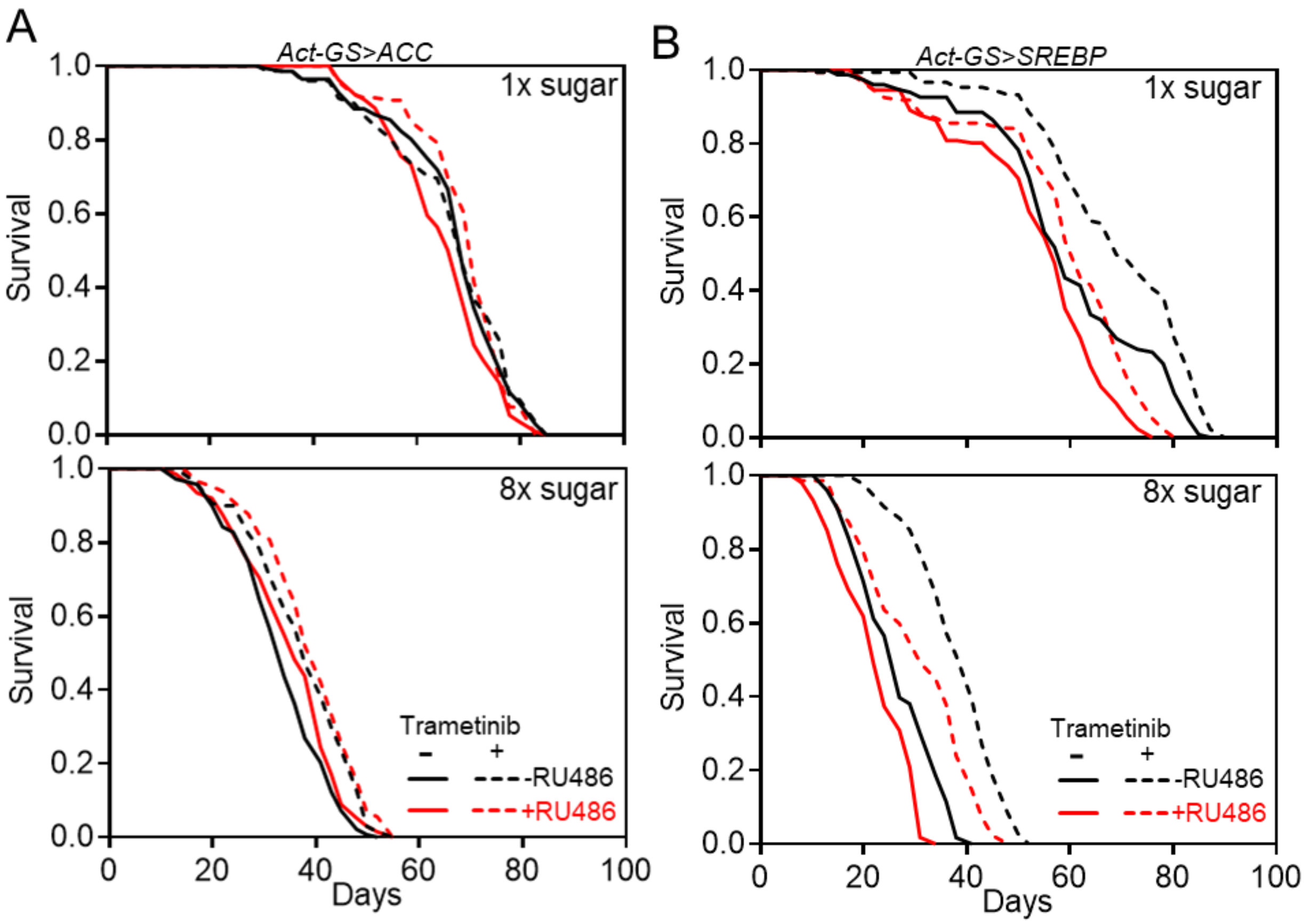

Supplementary Figure S2. Effects of trametinib on the survival of flies expressing additional *ACC/SREBP* transgenes, related to Figure 3.

(A) Survival of female *Act-GS>UAS-ACC (Bloomington stock #63225)* flies on normal sugar (1x) or high sugar (8x) either in the presence (red lines) or absence (black lines) of RU486 and either vehicle alone (sold lines) or trametinib (dotted lines). (1x sugar, -RU486 -trametinib n =125 dead/20 censored, -RU486 +trametinib n =137 dead/13 censored, p = 0.775, Log-rank test; +RU486 -trametinib n =125 dead/8 censored, +RU486 +trametinib n =128 dead/12 censored, $p = 2.2 \times 10^{-3}$, Log-rank test; 8x sugar, -RU486 -trametinib n =138 dead/4 censored, -RU486 +trametinib n =141 dead/9 censored, $p = 1.1 \times 10^{-5}$, Log-rank test; +RU486 -trametinib n =135 dead/4 censored, +RU486 +trametinib n =145 dead/5 censored, $p = 2.4 \times 10^{-4}$, Log-rank test). (B) Survival of female *Act-GS>UAS-SREBP (Bloomington stock #41018)* flies on normal sugar (1x) or high sugar (8x) either in the presence (red lines) or absence (black lines) of RU486 and either vehicle alone (sold lines) or trametinib (dotted lines). (1x sugar, -RU486 -trametinib n =140 dead/10 censored, -RU486 +trametinib n =139 dead/11 censored, $p = 2.8 \times 10^{-7}$, Log-rank test; +RU486 -trametinib n =139 dead/11 censored, +RU486 +trametinib n =140 dead/10 censored, $p = 1.1 \times 10^{-5}$, Log-rank test; 8x sugar, -RU486 -trametinib n =128 dead/20 censored, -RU486 +trametinib n =116 dead/21 censored, $p = 4.8 \times 10^{-5}$, Log-rank test; +RU486 -trametinib n =129 dead/21 censored, +RU486 +trametinib n =132 dead/14 censored, $p = 1.9 \times 10^{-11}$, Log-rank test).

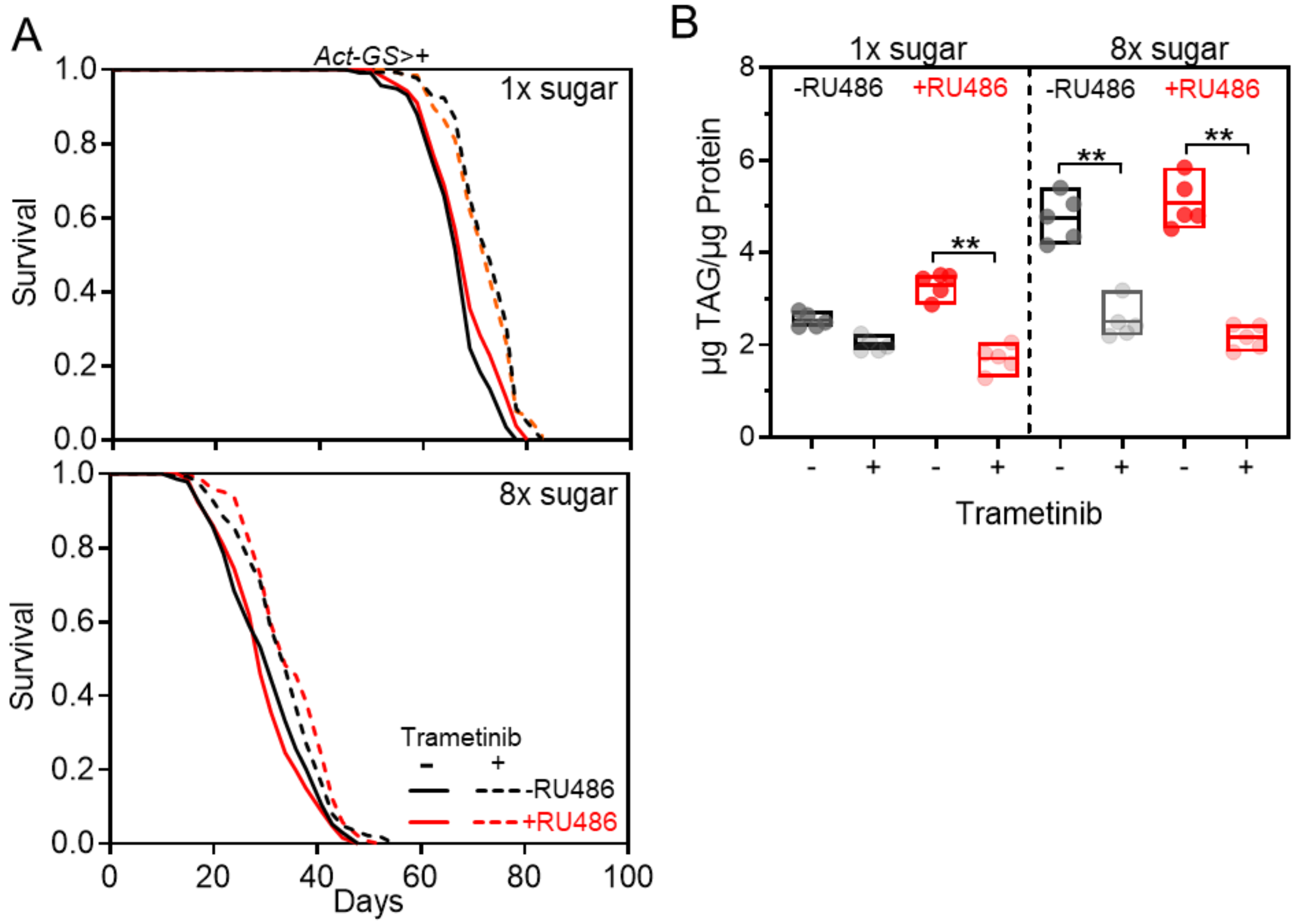

Supplementary Figure S3. Effects of trametinib on survival and TAG storage in *Act-GS* controls, related to Figure 3.

(A) Survival of female *Act-GS*>+ flies on normal sugar (1x) or high sugar (8x) either in the presence (red lines) or absence (black lines) of RU486 and either vehicle alone (sold lines) or trametinib (dotted lines). (1x sugar, -RU486 -trametinib n =100 dead/21 censored, -RU486 +trametinib n =113 dead/28 censored, p = 9.3 x 10^{-14}, Log-rank test; +RU486 -trametinib n = 101 dead/26 censored, +RU486 +trametinib n = 101 dead/35 censored, p = 1.5 x 10^{-5}, Log-rank test; 8x sugar, -RU486 -trametinib n =137 dead/2 censored, -RU486 +trametinib n =133 dead/5 censored, p = 6.9 x 10^{-3}, Log-rank test; +RU486 -trametinib n =126 dead/19 censored, +RU486 +trametinib n =142 dead/2 censored, p = 5.8 x 10^{-7}, Log-rank test). (B) TAG concentrations in homogenates from 10-day old female *Act-GS*>+ flies maintained on normal (1x) or high (8x) sugar diets either in the presence (red) or absence (black) of RU486 containing either vehicle alone (-) or trametinib (+). Box plots represent minimum, mean and maximum values with individual biological replicates overlaid as points (n = 5 biologically independent samples). ** p < 0.05, ANOVA with post-hoc Tukey's HSD.

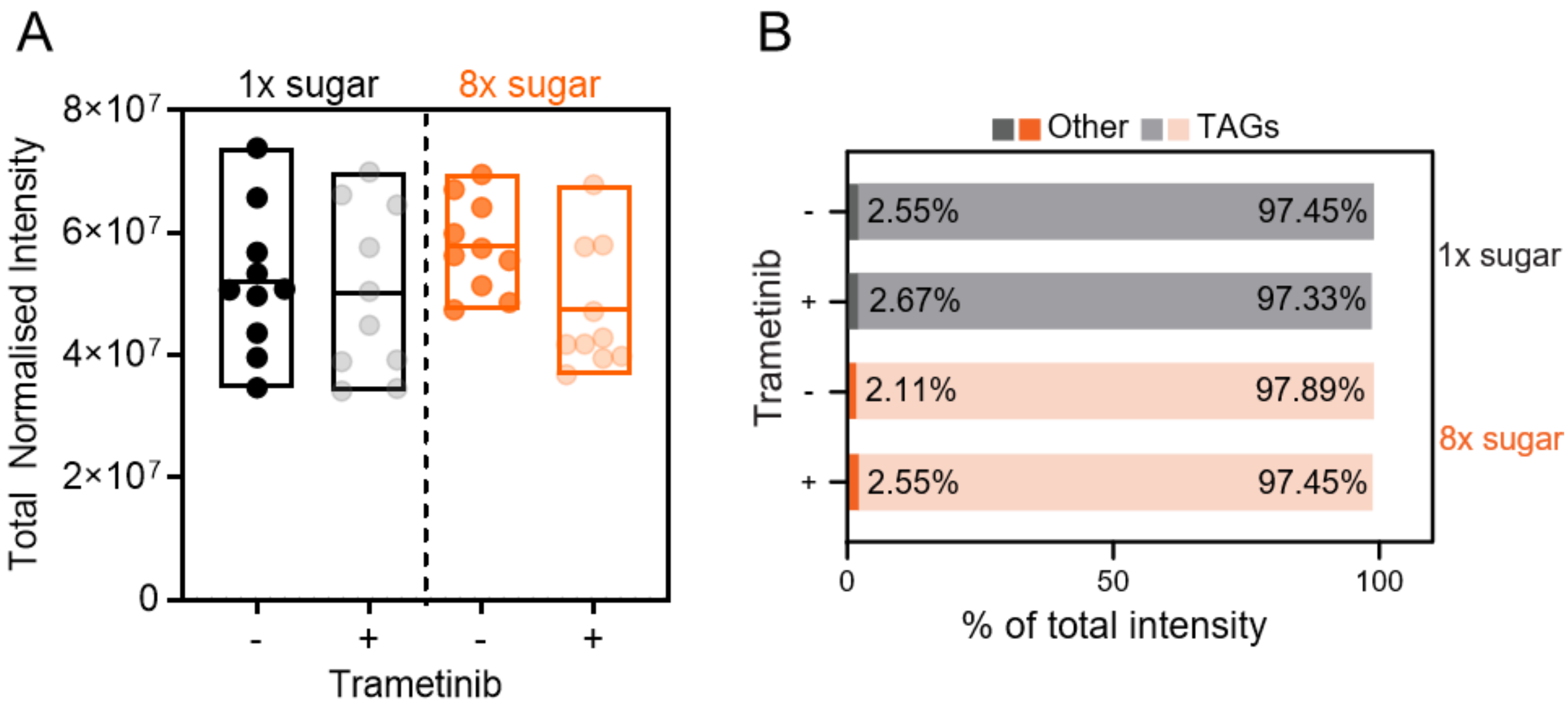

Supplementary Figure S4. Contribution of Triaceylglycerides (TAGs) to the whole lipidome in *Drosophila* extracts, related to Figure 4.

(A) Total normalised intensities from LC-MCMS analysis of total TAGs in whole fly lipid extracts prepared from 10-day old females maintained on normal (1x) or high (8x) sugar diets either in the presence (+) or absence (-) of trametinib (+). Box plots represent minimum, mean and maximum values with individual biological replicates overlaid as points (n = 10 biologically independent samples). (B) Average % of total intensity values attributed to TAGs or all other lipid species present in whole fly lipid extracts after feeding on normal (1x) or high (8x) sugar diets either in the presence (+) or absence (-) of trametinib (+) (n = 10 biologically independent samples).

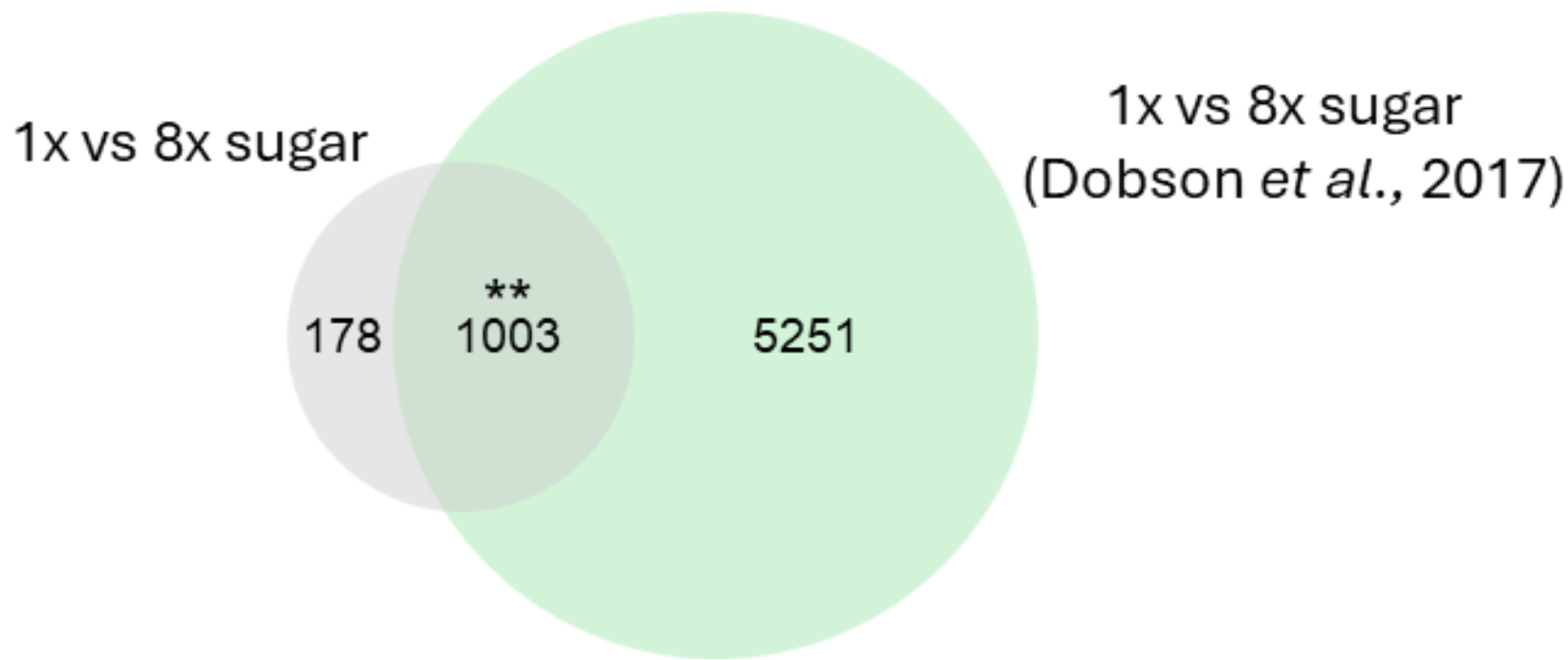

Supplementary Figure S5. Differential gene expression analysis of flies fed with a high sugar diet, **related to Figure 5.**

Proportional Venn diagram comparing the differentially expressed genes in *wDah* female flies exposed to 8x sugar diet vs 1x sugar diet to those of *wDah* females exposed to a similar dietary protocol as reported in (Dobson et al., 2006). Numbers indicate the number of genes in each category. 1003 genes were similarly differentially expressed in both data sets and this overlap was highly significant (** $p < 1.59 \times 10^{-116}$, hypergeometric distribution).

Condition	Median Lifespan (Days)	n Dead	n Censored	% Change	P-value (Log rank) vs control	Control
1x sugar, Trametinib -	74.5	145	1			*
1x sugar, Trametimib +	81.3	145	5	9% ↑	5.18×10^{-07}	
8x sugar, Trametinib -	32.6	144	4			*
8x sugar, 8x Trametinib +	39.6	133	3	21% ↑	1.01×10^{-07}	

Table S1. Survival data of flies fed on a high sugar diet in both the absence and presence of trametinib, related to Figure 1.

Details	Coefficient	Coefficient (estimate)	exp (coeff)	SE (coeff)	z	p
Dead = 567<br>Censored = 13<br><br>Effects of sugar concentration and trametinib treatment analysed using a priori contrasts.	sugar	3.823700	45.773239	0.236773	16.149	$< 2 \times 10^{-16}$
	trametinib	-0.590037	0.554307	0.119960	-4.919	8.72×10^{-7}
	sugar : trametinib	0.005148	1.005162	0.171598	0.030	0.976

Table S2. Cox proportional hazards analysis of survival data, related to Figure 1.

Coefficient estimate is the logarithm of the relative risk, where a positive value indicates an increase in relative risk of death. Total flies dead = 567, censored = 13.
